# Dr. Hodgkin's Catalogue of the Preparations in the Anatomical Museum of Guy's Hospital

**Published:** 1830-10-01

**Authors:** 


### VIII.

A Catalogue of the Preparations in the Anatomical Museum of Guy's Hospital. Arranged and Edited, by desire of the Treasurer of the Hospital, and of the Teachers of the Medical and Surgical School, by *Thomas Hodgkin*, M. D. Licentiate of the Royal College of Physicians of London; Demonstrator of Morbid Anatomy, and Curator of the Museum at Guy's Hospital; and Member of various learned Societies. Octavo, London, 1829. Pp. 598.

That the study of pathological anatomy, which formerly was somewhat neglected in this country, has of late years much increased, may be inferred from the following sentence in the Introduction of the volume now submitted to our examination.

"The author at one time proposed to take the Morbid Anatomy of Dr. Baillie, as the text-book for the Museum, and to have placed the preparations in accordance with the arrangement adopted in that work; but he very soon abandoned this design, finding the work inadequate to the purpose." *Introd. p.* ix.

This good effect, in common with many others, is in a great measure attributable to the general peace which has happily prevailed during the last fifteen years, and to the free intercourse thereby afforded with the continental schools; in several of which this branch of science has long been cultivated under peculiar advantages, and with proportional zeal and success.

But, as it is a common fault with mankind to pass from one extreme to its opposite, there may, perhaps, be a tendency in some persons to over-rate the value of pathological anatomy, and to relax in their attention to other departments of the healing art, which are, at least, of equal importance. It ought, however, fairly to be admitted that, without an intelligent regard to the phenomena of living action, and to the operation of agents, both salutary and noxious, a mere knowledge of the internal results of disease would be of little avail; although, without that knowledge, medical science would undoubtedly be incomplete.

The advantages derived from this source are not the less certain because they are partly negative and indirect; consisting, either in a clearer illustration of healthy structure and function, or in a more exact definition of the limits of our acquaintance with disease, and of our power of controlling it. From inspecting the bodies of those who die of tetanus, or of hydrophobia, we learn, for example, that the most violent and fatal disorders are not necessarily attended with visible lesion. From observing the ravages of unsubdued inflammation, we are admonished to avoid indecision and delay, during the only period when remedies can be successfully employed; and, from perceiving the incurable nature of some organic diseases, we are instructed to abstain from useless or pernicious interference; and to confine our practice to those measures which tend to husband the strength, or to promote the tranquillity of the system.

Among the more positive benefits resulting from this study, we need only mention the judicious treatment of continued fever, dictated by a knowledge of the intestinal ulceration, which dissection has proved so often to accompany that disorder, but which, without its aid, might for ages have remained unsuspected ; and yet, to this single discovery is probably annexed the gratifying prospect of an extensive preservation of human life.

Admitting, therefore, the importance of pathological anatomy when rightly applied, it is obvious that the private practitioner, engaged in the arduous duties of his profession, has, in general, but limited means for its cultivation ; and that, for its effective advancement and diffusion, large hospitals alone furnish adequate materials and opportunities. Without the dexterity and sagacity acquired by experience, without a just method, deliberate and universal inspection, and accurate records, the study is liable to much fallacy and imperfection ; and should, therefore, be prosecuted by some at least who, possessing suitable aids and qualifications, are enabled to devote much of their time and attention to its improvement.

Owing to various causes, which it is not necessary here to specify, the peculiar advantages afforded by large hospitals for the augmentation of medical science have not always been rendered so available as could have been wished. In the present day the case is happily otherwise; and, without intending any invidious distinction, it is a matter of just congratulation that, in the school of Guy's Hospital, as well as in several others, this object is now pursued with equal zeal and intelligence.

The merit of establishing the museum of this hospital must, in the first place, be ascribed to its treasurer, Benjamin Harrison, Esq. whose exertions during a long series of years to promote the usefulness of the institution, both as an asylum for the sick, and as a school of medicine, are generally known and appreciated. Under his direction, the ample funds of the hospital permitted the erection, about five years since, of a spacious building, entirely devoted to scientific purposes; comprising a commodious lecture-room, apartments for dissection, and the anatomical museum, of which the descriptive catalogue is now under our consideration.

But, in such an undertaking, the most able director, even with funds and materials at his disposal, could not accomplish much, without the aid of well-qualified agents; and, in this respect, the institution is fortunate in having secured the co-operation of the author of this work ; whose character, both professional and personal, stands too high to admit of eulogium.

In presenting a short analysis of Dr. Hodgkin's Catalogue, we shall, as far as possible, avail ourselves of its contents ; conceiving that, by exhibiting fair specimens of its materials, and of the manner in which they are proposed, we shall do the fullest justice both to the author, and to the reader.

Respecting the facilities afforded at Guy's Hospital for the cultivation of morbid anatomy, it is observed :—

"Where, as in this hospital, the patients are admitted without reference to individual interest, but by a superiority of claim, founded solely on the greater severity and urgency of their particular cases, it follows, as a necessary consequence, that the average of interesting cases must be particularly high. Some idea of the ample field for pathological anatomy presented at Guy's Hospital may be formed, from the following statement of the mortality which has taken place in the institution during the three last years."

From this statement it appears that the annual average of deaths, between the years 1825 and 1828, was 281; and that the number of beds at present devoted to patients amounts to 421.—*Introd. p.* iv. As a proof of the activity with which the museum has latterly been augmented, chiefly by the exertions, or under the direction of the author, we are informed :—

"It is only from this period [namely, within the last four or five years] that the departments of descriptive and comparative anatomy can be said to date their existence. The department of morbid anatomy has, likewise, been greatly enriched, not only by the internal resources of the establishment, but also by the donations of numerous contributors from without. In fact, with the exception of a nucleus of scarcely five hundred preparations, the whole collection, at present amounting to upwards of three thousand specimens, has been formed within the short space of four years."—*Introd. p.* v. vi.

It ought not, however, to be concealed that, for the production and preservation of this nucleus, under circumstances far less favourable than those now enjoyed, the institution is chiefly indebted to the industry and ability of its resident medical officer, Richard Stocker, Esq.

Among the principal contributors to the museum, we find, as might naturally have been expected, the present and some of the former physicians, surgeons, and pupils of the hospital, the treasurer, the Royal Veterinary College, and the author himself. A considerable number of specimens has been obtained from the well-known collection of Joshua Brookes, Esq. and some of the most beautiful and splendid have been supplied by the hands of Sir Astley Cooper.

"For the illustration of the structure and diseases of the teeth, the museum possesses the collection of the late Joseph Fox, enriched by many valuable and curious additions from his able successor, Thomas Bell.

The department of casts and models forms too important a feature in the museum to be left unnoticed. In this department, youthful as is the museum, it is, perhaps, not too much to say that it yields to none in this country. Its advantageous position in this respect must be attributed to the fortunate circumstance of the treasurer's having attached to the service of the hospital Joseph Towne, an artist who has the signal merit of having both created his art for himself, and arrived at such a proficiency in it, that his works, already very numerous, rival, if not surpass, those of the best and most distinguished masters of Florence, and Bologna.

The drawings, and diagrams, although not introduced into the present catalogue, must not be omitted in the enumeration of what has been done at Guy's Hospital to facilitate the communication of pathological knowledge. The spirited and accurate pencil of C. J. Canton, constantly employed in this department for the service of the hospital, by preserving the recent colours and appearances of diseased parts, forms an invaluable supplement to the wet preparations, which, after the most successful efforts, must often fail in retaining any thing beyond the form and texture."—*Introd. p.* vi. vii.

But all these advantages would have been of little avail, either to the hospital pupil, or to the professional visitor, without a good catalogue. To an intelligent student, in pursuit of knowledge rather than of amusement, nothing can be more unsatisfactory than the common practice of sauntering through a large museum, without an adequate knowledge of its contents. A competent living guide is not always at hand; and, even when procurable, it is often far more convenient to obtain the requisite information from a well-composed manual, by the aid of which any part of the collection may be inspected at pleasure, and the reflexions of the observer may be pursued without hurry or interruption.

Such, then, is the nature of the volume now before us; a volume which is by no means, as the title might seem to import, a mere catalogue or inventory, but of which, in addition to a condensed and perspicuous description of the preparations themselves, nearly a fourth part consists of interesting observations on the several branches of the science; the greater number furnished by the author, and a few derived from other valuable sources. After an historical introduction, from which extracts have already been given, and three useful tables, representing the general appearances observed on the inspection of bodies, the principal organic deviations from the healthy state, and the order of arrangement adopted in the work, the volume consists of three grand divisions, describing the preparations illustrative of healthy, morbid, and comparative anatomy.

The latter part, classified according to the system of Cuvier, contains many beautiful specimens; among others, several subservient to veterinary surgery, and one of the best articulated skeletons of the female elephant to be found in the country: but this part, being as yet in its infancy, occupies the smallest space in the catalogue. It is, however, enriched by a manual of directions respecting the collection, preservation, and packing of objects of natural history, abstracted from the official instructions of the professors of the Royal Garden at Paris.

The two principal departments, namely, those of sound and morbid human anatomy, are subdivided into the following sections, which we here subjoin; because, although for different purposes different plans may be required, the arrangement adopted on this occasion appears highly judicious, and may be imitated with advantage in other similar institutions. In both departments these sections occur in the ensuing order.—Bones—soft parts about the bones—vascular or circulatory systems—nervous system and organs of the senses—vocal and respiratory organs—digestive organs—urinary organs—genital organs of the female—genital organs of the male—and utero-gestation. Besides these sections, which are common to both, the first part includes a section illustrative of the fluids, and another of miscellaneous objects; while the second part adds a section of diseases of the peritoneum, another of parasitical animals, and a third of models and casts. To each of these divisions are prefixed, as has been already noticed, introductory remarks, tending to fix the attention of the reader on the most important points, and to prepare him for inspecting with advantage the various objects presented to his observation.

Concerning the form in which the catalogue is ultimately disposed, the following explanation is given by the author.

"In printing the catalogue, the tabular form has been chosen, as the most convenient for reference, and, at the same time, the most concise and intelligible. In the first column is placed the number which refers to the preparation. In the next is the description of the preparation. This, though in general necessarily short, is sufficient to point out the object which the specimen is designed to illustrate. When the preparation is of more than usual interest, the description is given at greater length. The next column contains a reference to the fuller details of the case. The greater number of these references are made to the manuscript histories of the hospital cases and inspections, of which there are now thirteen volumes, most of which have been collected in the course of the last three years. In the same column are placed references to printed books, when the preparations have been described or alluded to in published works. When the preparation has been acquired as a gift, reference is made in this column to the donor's account of the case, if such a document accompanied the preparation. The last column shews the source whence the preparation was derived, and records the names of those whose liberality has enriched the collection."—*Introd. p.* xiv. xv.

That portion of the catalogue which is devoted to healthy anatomy is not only useful in itself, but also eminently conducive to the study of the larger section of morbid anatomy which immediately follows, or rather the two branches mutually illustrate each other; since the knowledge, both of structure and of function, is best promoted by alternately viewing them in their natural state, and in all their various modes of irregularity and aberration. Among the original dissertations inserted in this part of the work, is a copious extract from an unpublished memoir on the anatomy of the brain, presented to the French Academy of Sciences by Dr. Foville, of Rouen, who, with peculiar opportunities of research, has for some years past directed his attention to this difficult and interesting subject. Besides quotations from Dr. Hodgkin's inaugural dissertation on absorption, at Edinburgh, in 1823, and from his earlier treatise on the use of the spleen, the remainder of the introductory remarks are chiefly derived from observations made by the author, and J. J. Lister, Esq. with the aid of an achromatic compound microscope of superior power, on the component particles of the animal solids and fluids. In reference to the solids, it hence results that their ultimate texture, instead of being globular, as has hitherto been supposed, is either irregularly granular, as in the brain and in some parenchymatous parts, or fibrous, as in most other cases.

In the description given by our authors of the particles of the blood, the reports of former observers are, in like manner considerably modified, as will appear from the following extracts.

"We have viewed the particles, both wet and dry, both as opaque, and as transparent objects, under every variety of power and light; and we lay no stress on observations which have not been confirmed by frequent repetition. To us the particles of human blood appear to consist of circular, flattened, transparent cakes, which, when seen singly, appear to be nearly, or quite, colourless. Their edges are rounded; and, being the thickest part, occasion a depression in the middle, which exists on both surfaces. * * * The concavity of the disks is, however, extremely trifling; and, under particular circumstances, in a few of the particles, the surface is to all appearance quite flat. Notwithstanding the great uniformity in the size of the particles of the blood, so long as they retain unimpaired the form which they possess on escaping from the body, their real magnitude has been so variously estimated that we judged it worth while to attempt a new measurement. In doing so, we adopted a method somewhat different from those hitherto employed. A camera lucida is adapted to the eye-piece of the microscope in such a manner that, the distance of the paper being ascertained, the object may be drawn on a known scale. Tracings of several of the images being made, they were applied to, and compared with, the images of other particles, until their accuracy was established. The diameter of the particles obtained in this manner may be pretty correctly stated at the three-thousandth part of an inch. * * * The thickness of the particles, which is, perhaps, not so uniform as the diameter of the disks, is, on an average, to this latter dimension as 1 to 4.5." * * * The particles of pus "appear to be as irregular in size and figure as those observed in the brain, and bear no resemblance to those of the blood." * * * The particles of milk "appear to be perfect globules; but, far from being uniform, they present the most remarkable varieties in respect to size. Whilst some are more than double, others are not a tenth part of the size of the particles of the blood, to which they bear no resemblance."—*Observ. on Sec.* XI. *of Part* I.

Until these observations have been repeated by others, they scarcely fall within the province of critical decision; yet it must be admitted that they have been made under favourable circumstances, and apparently with much care and dexterity, by observers well acquainted with the labours of their predecessors, and well disposed to afford each other correction and assistance.

The larger and more important part of the museum, which occupies the second and principal place in the catalogue, is, like the first, divided into sections, corresponding to the various systems of the body; for which the author acknowledges himself considerably indebted to the excellent anatomical work of Professor Meckel, and for the adoption of which he gives the following satisfactory reason.

"The habit of frequently reviewing, in the same succession, preparations brought together for the purpose of illustrating the pathology of a particular organ, or apparatus, cannot fail to render considerable practical assistance to diagnosis, by enabling the memory rapidly to bring under review the various possible alterations with which the organs suspected of disease may be affected; and, whilst we make the choice of that to which the united symptoms appear most decidedly to point, we may avoid the danger of overlooking the right one through inadvertence or forgetfulness. For this reason, it has been thought better to arrange the specimens in the museum under the heads of particular systems, or apparatus, rather than under those of the elementary tissues."—*Introd. p.* x.

To give the reader an adequate notion of a collection of morbid anatomy comprising more than three thousand preparations would, of course, be a vain undertaking; nevertheless, some of its more remarkable contents may be briefly noticed. Among diseased bones, we find two instances of the processus dentatus so much enlarged as, by its pressure, to have occasioned paralysis;—examples of a singular spongy degeneration of the cranial bones;—several cases of fungoid disease;—a fracture of the neck of the humerus, with dislocation of the head of the bone;—and a complete anchylosis of the lower jaw. Among morbid appendages of bones, are observed fatty degenerations of muscles;—and that remarkable inflammation of synovial membranes which occasions absorption of the articular cartilages. The diseases of the vascular system include rare specimens of scrofulous tubercles, true scirrhus, and fungoid tumours, imbedded in the substance of the heart;—also, several examples of retroversion of the aortic valves; a derangement of structure which, with the exception of a slight incidental notice by Bertin, seems to have been first described by Dr. Hodgkin. The section devoted to diseases of the digestive organs contains some good specimens of that ulceration of the mucous glands of the intestines which, of late years, has so often been observed to attend continued fever. It also presents examples of malignant disease in the stomach and œsophagus;—fungoid and melanotic tubercles in the liver;—deficiency of the gall-bladder;—uncommon degenerations of the pancreas and spleen;—and, as a matter of curiosity, the stomach of John Cuming, who died, in Guy's Hospital, ten years after having swallowed a considerable number of knives, most of which he retained till the time of his death.

"Amongst the preparations of diseased kidneys, none will be viewed with more interest than those which illustrate the valuable observations of Dr. Bright, respecting that remarkable, though previously undescribed, mottling degeneration of these organs, which he has shewn to be frequently, though by no means invariably, accompanied by a disposition to dropsical effusion," but which is always attended with an "albuminous condition of the urine, as shewn by the application of heat. * * * This degeneration appears more particularly to affect the cortical part."—*Obs. on Sect.* VII. *of Part* II.

There are, likewise, several examples of malignant disease of the kidneys and bladder; cases of congenital deficiency of the anterior part of the bladder; and a large collection of urinary calculi, arranged according to the method of Dr. Prout.

Omitting, for the sake of brevity, the diseases of the sexual organs, we arrive at those of the peritoneum, concerning which the author justly remarks:—

"The subject of hernia is the most important and peculiar which belongs to the morbid anatomy of the peritoneum. Although the museum does not at present possess a very considerable number of preparations relating to this subject, the student will, nevertheless, find that they illustrate some of the most curious and important points connected with it; and he is particularly invited to examine them, in conjunction with the splendid and valuable work of Sir Astley Cooper, now greatly enriched by its editor, C. Aston Key."—*Obs. on Sect.* X. *of Part* II.

Under the head of morbid utero-gestation, are some remarkable instances of real or supposed hermaphrodites. Respecting malformation in general, it is well observed by Dr. Hodgkin,—

"The subject of malformation and monstrosity is one of the most interesting to which the attention of the physiologist can be directed. Cases of monstrosity may be regarded as invaluable experiments, conducted for us by Nature herself, by which she seems to give us a little insight into some of the laws which appear to regulate the formation and development of animal beings."—*Observ. on Sect.* XI. *of Part* II.

Although the author does not undertake to transfer to this branch of the work the substance of his Lectures on Pathological Anatomy, he has enriched it, as well as the former, with interesting observations prefixed to the several sections, and furnishing a useful introduction to their contents. The principal of these are on bronchocele, on certain adventitious structures, and on irregular productions in the ovaries, and in other parts.

On the subject of bronchocele reports are collected from almost every quarter of the globe, including some made by the author himself, when travelling among the Alps. He justly concludes that snow-water, abstractedly considered, cannot be the cause of this singular deformity; since it is common where snow is unknown, and rare in some countries where scarcely any other water is procurable. His own conjecture, that it is attributable to water holding in solution certain salts of lime, although, perhaps, an approximation to the fact, is, on similar grounds, liable to doubt; for it would be as difficult to prove such an impregnation among the granite rocks where the disease so frequently abounds, as to find a case of goitre in various districts where the waters are decidedly calcareous. A still later observer, Mr. Thomas Colbeck, plausibly endeavours to trace the disease to the use of turbid water; such as, in mountainous countries is, indeed, usually the state of water obtained from snow; but, admitting the probability that impure water of some kind is the principal cause of bronchocele, the precise nature of the noxious ingredient seems hitherto to have eluded discovery.

On the very curious subject of the irregular formation in the ovaries, and in other parts, of cysts, containing teeth, fat, hair, &c. the author examines the opinions of several of the most eminent physiologists; by some of whom these singular aberrations are regarded "as calculated to throw light on phenomena the most obscure, and, at the same time, the most stupendous."—*Observ. on Sec.* VIII. *of Part* II. To offer a decision on this abstruse point, the narrowness of our limits, as well as the difficulty of the investigation, at present forbids; and, for similar reasons, we shall merely notice the author's original and ingenious views respecting the production of several adventitious morbid structures, and which are more fully developed in his dissertation, inserted in the Medico-Chirurgical Transactions, Vol. XV. We shall only observe, that these views seem to have resulted from the patient and accurate dissection of a great number of cases, and promise to elucidate a very interesting and difficult branch of pathology.

Our analysis of this work must not conclude without again noticing a very important part of the museum; namely, the collection of models and casts, already comprising nearly three hundred specimens, and forming a most valuable supplement to the original preparations, to which, as being perfect and unchangeable, they are, indeed, often to be preferred. Not confined to the representation of morbid appearances in the dead body, they include, also, that of cutaneous diseases, tumours, ulcers, fractures, and dislocations; displaying all the characters which these complaints severally present during life, excepting only those of danger and disgust. No verbal description, however graphic; no drawing, however spirited, can equal the exquisite imitation of natural form and colour in these fac-simile copies. By their aid, the student is enabled, in the course of a few hours, and with the utmost personal convenience, to acquire a familiarity with the outward aspect of many remarkable accidents and disorders, of which, without a serious expense of time, labour, and pecuniary resources, he can rarely hope to study the originals. In the department of sound anatomy, several delicate dissections of principal or complicated organs, such as the brain and its nerves, the internal ear, the muscles, tendons, and ligaments of the hand and foot, the gravid uterus and its contents, and the parts concerned in hernia, are thus represented in the most accurate and perspicuous manner. It is pleasing to repeat, what has already been noticed, that many of the best of these imitations have been executed by a native artist, Joseph Towne, Esq. the successful rival of the celebrated modellers of Florence, and Bologna, to whom, for a long time, this ingenious art seemed to be exclusively confined.

We now take leave of the author, with the highest recommendation of his instructive and interesting volume. To the student who has the privilege of visiting the museum of Guy's Hospital it must prove an invaluable guide; and even to the distant reader it will communicate much useful knowledge, in a pleasing and methodical manner. The indefatigable labour and various talent, requisite for preparing and completing this work, can be appreciated by those alone who have engaged in such undertakings. Unlike most other books, it does not merely suggest opinions, or describe facts, but furnishes an introduction to a vast number of original and important objects, accompanied, for the most part, with that subsidiary information, by means of which they may be inspected with intelligence and advantage. Such a work appears in this country almost for the first time; and, as it has at once attained a high degree of perfection, it may confidently be expected that it will not only promote the progress of pathological anatomy in the school where it originated, but still more extensively, by exciting the honourable emulation of other similar institutions.

